# Tropism, intracerebral distribution, and transduction efficiency of HIV- and SIV-based lentiviral vectors after injection into the mouse brain: a qualitative and quantitative in vivo study

**DOI:** 10.1007/s00418-017-1569-1

**Published:** 2017-04-10

**Authors:** Juraj Hlavatý, Zbyněk Tonar, Matthias Renner, Sylvia Panitz, Helga Petznek, Matthias Schweizer, Silke Schüle, Björn-Philipp Kloke, Rudolf Moldzio, Kirsti Witter

**Affiliations:** 1 Department of Pathobiology, Institute of Anatomy, Histology and Embryology, University of Veterinary Medicine Vienna, Veterinärplatz 1, 1210 Vienna, Austria; 2 Faculty of Applied Sciences, European Centre of Excellence NTIS, University of West Bohemia in Pilsen, Univerzitní 8, 306 14 Pilsen, Czech Republic; 3 Department of Medicinal Biotechnology, Paul-Ehrlich-Institut, Langen, Germany; 4 Institute of Virology, University of Veterinary Medicine, Vienna, Austria; 5 Institute of Medical Biochemistry, University of Veterinary Medicine, Vienna, Austria

**Keywords:** Gene transfer, Double immunofluorescence, EGFP, Vpr, Vpx, Stereology

## Abstract

Lentiviruses are suitable to transfer potential therapeutic genes into non-replicating cells such as neurons, but systematic in vivo studies on transduction of neural cells within the complete brain are missing. We analysed the distribution of transduced cells with respect to brain structure, virus tropism, numbers of transduced neurons per brain, and influence of the Vpx or Vpr accessory proteins after injection of vectors based on SIVsmmPBj, HIV-2, and HIV-1 lentiviruses into the right striatum of the mouse brain. Transduced cells were found ipsilaterally around the injection canal, in corpus striatum and along corpus callosum, irrespective of the vector type. All vectors except HIV-2SEW transduced also single cells in the olfactory bulb, hippocampus, and cerebellum. Vector HIV-2SEW was the most neuron specific. However, vectors PBjSEW and HIV-1SEW transduced more neurons per brain (means 41,299 and 32,309) than HIV-2SEW (16,102). In the presence of Vpx/Vpr proteins, HIV-2SEW(Vpx) and HIV-1SEW(Vpr) showed higher overall transduction efficiencies (30,696 and 27,947 neurons per brain) than PBjSEW(Vpx) (6636). The distances of transduced cells from the injection canal did not differ among the viruses but correlated positively with the numbers of transduced neurons. The presence of Vpx/Vpr did not increase the numbers of transduced neurons. Parental virus type and the vector equipment seem to influence cellular tropism and transduction efficiency. Thus, precision of injection and choice of virus pseudotype are not sufficient when targeted lentiviral vector transduction of a defined brain cell population is required.

**Electronic supplementary material:**

The online version of this article (doi:10.1007/s00418-017-1569-1) contains supplementary material, which is available to authorized users.

## Introduction

Many neurodegenerative diseases such as Alzheimer’s, Parkinson’s, and Huntington’s disease still can only be treated inadequately. Hence, novel treatment modalities are desperately needed. Gene therapy could hold the promise of therapeutic progress (Blömer et al. 1996; Wong et al. 2006; Eleftheriadou and Mazarakis 2015). As many neurodegenerative diseases develop with progressive, incurable degeneration of neuronal cells, gene therapy approaches for the treatment of such disorders require vector systems that can efficiently transduce non-dividing neurons. Lentiviral vectors have been shown to do so and by integrating their genome into the genome of the transduced cells they supposedly provide permanent expression of the transferred therapeutic gene in these cells and also in the progeny of transduced dividing neuronal precursors (Lundberg et al. 2008). In addition to these therapeutic approaches, lentiviral vectors can be used for modelling of neurodegenerative disorders in different experimental settings (Déglon and Hantraye 2005; Eleftheriadou and Mazarakis 2015).

Vectors derived from human immunodeficiency virus 1 and 2 (HIV-1- and HIV-2) together with those derived from simian immunodeficiency virus (SIV), equine immune anaemia virus (EIAV), or adeno-associated virus are among the most intensively studied systems used for gene transfer into neuronal cells (Mitrophanous et al. 1999; D´Costa et al. 2003; Liehl et al. 2007; Trabalza et al. 2013; Hocquemiller et al. 2016; Stewart et al. 2016). Data from the first clinical trials (phase 1 or 1/2) utilising adeno-associated and lentiviral vectors for therapy of brain diseases are available (Mittermeyer et al. 2012; Palfi et al. 2014). Although a number of different vectors have been proven to be suitable for neuronal gene transfer, our study concentrates primarily on SIV-derived and closely related HIV-derived vectors. Our intention emanated from the fact that SIVsmmPBj-derived vectors were able to infect quiescent primary monocytes, dendritic cells as well as other G_0_-arrested cells (Mühlebach et al. 2005; Wolfrum et al. 2007; Grabski et al. 2011); however, their suitability for neuronal infection has not been shown yet. Based on the similarities in virus genome design and presence of accessory genes, we have chosen vectors derived from HIV-1 and HIV-2 as relevant standards, since all three tested viruses and vectors derived from them are closely related to each other and transduction of neuronal cells using HIV-derived vectors has been described previously (Naldini et al. 1996a; D´Costa et al. 2003).

In contrast to other retroviruses, pre-integration complexes of lentiviruses possess the unique ability to actively cross the nuclear membrane and thereby enabling infection of non-dividing or quiescent cells. The accessory proteins Viral Protein R (Vpr) and Viral Protein X (Vpx) unique to the HIV-1 and HIV-2/SIVsmmPBj/SIVmac lentiviruses, respectively, seem to be able to increase the success of transduction of G_0_ cells such as neurons or peripheral blood mononuclear cells. Different candidate mechanisms for this effect have been identified. The Vpx and Vpr accessory proteins are described to be directly involved in nuclear targeting (Singhal et al. 2006) and in translocation across the nuclear membrane (Heinzinger et al. 1994; Blömer et al. 1997; Pancio et al. 2000; Belshan et al. 2006), to increase viral gene transcription (James et al. 2016), and to facilitate infection by rescuing the viruses from a proteasome-dependent restriction pathway (Goujon et al. 2007).

So far, published in vivo studies on brain cell transduction after injection of transgenic lentiviruses concentrate mostly on single cell populations in defined regions of interest, such as corpus striatum and substantia nigra (Naldini et al. 1996a; Blömer et al. 1997; Bensadoun et al. 2000; Stewart et al. 2016), hippocampus (Naldini et al. 1996a; Blömer et al. 1997), or selected nuclei (Sinnayah et al. 2002). The possibility of external regulation of the transcription of lentiviral vectors incorporated into the genome of cerebral neurons in vivo has been shown, e.g., by Haack et al. (2004) and Vogel et al. (2004). To our knowledge, systematic in vivo studies on transduction of neurons and glial cells within the complete brain after intracerebral injection of transgenic lentiviral vectors have not been performed up to now.

The aim of our study was therefore to analyse the transduction potential of six vectors based on the SIVsmmPBj, HIV-2 and HIV-1 lentiviruses delivered stereotactically into the right striatum of the mouse brain by (1) assessing the distribution of transduced cells with respect to the anatomical structures of the brain; (2) identifying preferentially infected cell populations for each virus; (3) determining the absolute number of transduced neurons per brain after single, unilateral injection; and (4) finally, assessing the influence of the Vpx/Vpr accessory proteins on cell targeting, distribution, and number of transduced neurons.

## Materials and methods

### Animals

For cell cultures experiments, pregnant OF1/SPF mice at gestation day 13 were purchased from the Institute for Laboratory Zoology and Veterinary Genetics, Himberg (Austria). For in vivo studies, female C57Bl/6 mice obtained from the University of Veterinary Medicine, Vienna, Austria were used.

Animal experiments have been approved by the institutional ethics committee and Austrian government authorities (BMWF; BMWF-68.205/0225-II/10b/2008). Mice were kept and handled in accordance with the guidelines of the European Union Council (86/609/EU) for the use of laboratory animals.

### Viral vectors

Despite the different origins of the tested transfer vectors, their internal organization was similar. All vectors contained an enhanced green fluorescent protein gene (EGFP) driven by the spleen focus-forming virus (SFFV) internal promoter, the central polypurin tract (cPPT), the woodchuck hepatitis posttranscriptional regulatory element (WPRE), and a self-inactivating deletion in the 3´LTR. The structural proteins for virus particle assembly were provided from the respective packaging constructs. The accessory proteins Vpr and Vpx were present in the indicated vector particles. To ensure a broad host cell range of transduced cells, all vectors were pseudotyped with the envelope glycoprotein of the vesicular stomatitis virus (VSV-G). The transfer vector plasmids used for generation of HIV-1, HIV-2, or SIVsmmPBj vectors were pHIV-1-SEW, (Demaison et al. 2002), pHIV-2-SEW (Kloke et al. 2010), or pPBj-SEW (Kloke et al. 2010), respectively. Packaging constructs for generation of HIV-1, HIV-2, or SIVsmmPBj vectors were pCMVdR8_91 (Zufferey et al. 1999), pHIV-2d4 (Morris et al. 2004), or pPBj-pack (Kloke et al. 2010), respectively. For generation of vectors carrying the Vpr or Vpx accessory protein (vectors HIV-1SEW(Vpr), HIV-2SEW(Vpx), or PBjSEW(Vpx)), the expression constructs used were pVprHIV-1 (Schüle et al. 2009), pVpxHIV-2syn (Kloke et al. 2010), or pVpxPBjsyn (Kloke et al. 2010), respectively. In all cases, the envelope gene expression plasmid pMD.G2 coding for the VSV-G protein was used (Naldini et al. 1996b).

For generation of lentiviral vectors, 293T cells were co-transfected with the respective transfer vector plasmid, the respective packaging construct, the VSV-G expression construct, and an accessory protein expression construct if indicated. The resulting vector particles were either named PBjSEW, HIV-2SEW, and HIV-1SEW for vectors without the accessory proteins and PBjSEW(Vpx), HIV-2SEW(Vpx), and HIV-1SEW(Vpr) for those in which the Vpr/Vpx proteins were present. Vectors were purified and concentrated from cell culture supernatant by centrifugation through a sucrose cushion, and vector titres were evaluated by titration on HT1080 target cells in serial dilutions as described previously (Schüle et al. 2009).

### Preparation and infection of primary mesencephalic cell cultures

To assess the ability of the vectors to transduce brain cells, mouse primary mesencephalic cell cultures were infected with the vectors at a multiplicity of infection (MOI) of 1, and the EGFP expression was subsequently analysed 3 weeks later by immunohistochemistry. The primary mesencephalic cell cultures were prepared as described previously (Moldzio et al. 2006). Briefly, pregnant mice were sacrificed on gestation day 14, the brains of the embryos were dissected, and the ventral mesencephala were excised. After removal of the meninges, tissues were mechanically cut into small pieces in PBS (pH 7.2). Tissues were transferred into a sterile test tube containing 2 ml of trypsin (0.1%) and 2 ml DNase (0.02%) and incubated in a water bath at 37 °C for 7 min. After stopping the trypsin reaction with 2 ml of trypsin inhibitor (0.125 mg/ml) and centrifugation, the supernatant was discarded, and the remaining tissue pellet triturated three times. A cell suspension of 750,000 cells/ml was diluted with medium containing DMEM (87.4%), HEPES (8.7 mM), glucose (9.7 mM), glutamine (1.7 mM), penicillin and streptomycin (436.9 U/ml and 0.4 mg/ml), amphotericin B (2.2 µg/ml), and foetal bovine serum (8.7%). The dissociated cells were plated in poly-D-lysine-coated 4-well chamber slides (Nunc, Sigma, Vienna, Austria) (750 µl in each well; ~550,000 cells/well) and kept at 37 °C, 5% CO_2_ and 100% humidity. Medium was changed every second day and replaced after 6 days in vitro (DIV) with serum-free medium containing DMEM (94.4%), HEPES (9.4 mM), glucose (10.4 mM), glutamine (1.9 mM), penicillin and streptomycin (472.0 U/ml and 0.5 mg/ml), and B 27 supplement (1.4%).

At DIV 7, cells were infected with an equal amount of 5.5 × 10^5^ virus particles per well (MOI 1) in the absence of polybrene. During the experiment, medium was changed every second day. Three weeks after the infection, cells were washed with PBS, fixed using Accustain (Sigma, Vienna, Austria) for 20 min at room temperature (RT) and stained using antibodies against EGFP, NeuN, and GFAP as described earlier (Liehl et al. 2007). To identify the type of transduced cells in dissociated brain cultures, double immune-labelling was performed using the rabbit anti-EGFP antibody (1:2000) in combination with each of the following cell type-specific mouse antibodies: anti-glial fibrillary acidic protein (GFAP) (Chemicon International, Temecula, CA, USA 1:1000) antibody or anti-neuronal nuclear antigen (NeuN) antibody (Chemicon International, Temecula, CA, USA, 1:500), and, as secondary antibodies, either the Alexa Fluor 488 goat anti-rabbit IgG (Molecular Probes-Invitrogen, Paisley, UK, 1:100) or the Alexa Fluor 568 goat anti-mouse IgG (Molecular Probes-Invitrogen, Paisley, UK, 1:100), so that in all samples, EGFP appears in green, whereas the cell specific marker proteins appear in red. Samples were pretreated by microwaving in citrate buffer (pH 6.0; 4 × 5 min), washed in PBS, and blocked with 1.5% goat serum for 30 min at RT. Afterwards, they were incubated with anti-EGFP/anti-NeuN or anti-GFAP at 4 °C overnight, washed with PBS, and incubated with the secondary antibodies for 1 h at RT. Samples were again washed with PBS, a coverslip was placed over the sample, and it was examined with a confocal laser scanning fluorescence microscope with appropriate filter settings (Axiovision System, Zeiss, Vienna, Austria). In vitro transduction efficiency was estimated by counting transduced neurons (NeuN and EGFP double positive cells) in relation to the total amount of neurons (NeuN positive cells) in four randomly selected microscopic fields in two infection experiment replicas for each vector type.

### In vivo testing of the viruses

For comparison of the in vivo transduction capacity, adult C57Bl/6 female mice were used (*n* = 6 per virus type). For stereotactic surgery, animals were anesthetized by intraperitoneal injection of 10 mg ketamine/0.4 mg xylazine per 100 g body weight and placed in a small-animal stereotactic apparatus (Stoelting, Wood Dale, IL, USA) with the skull horizontal between lambda and bregma. After midline incision of the skin, a hole was drilled in the skull and 1 × 10^6^ of highly concentrated vector particles in a total volume of 5 µl were injected into the right striatum at a rate of 0.5 µl/min using a 26-gauge needle and a Hamilton syringe. Coordinates to target the striatum were 1 mm anterior, 2 mm lateral, and 3.5 mm ventral from the bregma, as measured from the skull surface according to Paxinos and Franklin (2003). After injection, the needle was left in place for additional 5 min before being slowly removed, the incisions were sutured, and animals were kept under observation until recovery. During the experiment, the mice were maintained with food and water available *ad libitum*. Three weeks after virus injection, mice were euthanized under anaesthesia by transcardial perfusion using 10 ml cold PBS and 10 ml Accustain solution (Sigma, Vienna, Austria). Brains were removed immediately from the skull and post-fixed by immersion in the Accustain fixative for at least 12 h at 4 °C. After paraffin embedding, tissues were further analysed.

### Histological processing of brains, immunohistochemistry, and microscopic analysis

All brains were embedded routinely in paraffin. They were cut exhaustively into serial sections at a section thickness of 18 µm. Sections were mounted on Superfrost™ slides coated with (3-aminopropyl)triethoxysilane (Sigma-Aldrich, Vienna, Austria), stained immunohistochemically, and analysed by confocal microscopy as follows.

To detect EGFP-positive neurons, an immunohistochemical double fluorescence staining using a NeuN antibody (NeuN, clone MAB377, Chemicon International, Temecula, CA, USA; neuronal marker) and a rabbit anti-GFP antiserum (Molecular Probes-Invitrogen, Paisley, UK) was performed. The sections were heated at 60 °C for 20 min to soften the paraffin and to enhance its removal. Still warm, they were deparaffinised in xylene, rehydrated in decreasing alcohol concentrations, and permeabilized in chilled acetone (−20 °C, 10 min). After washing in distilled water, the sections were transferred to phosphate buffered saline (PBS, pH 7.4). Unspecific binding activity was blocked with normal goat serum (DakoCytomation, Glostrup, Denmark). The sections were incubated overnight at 4 °C with a cocktail of both primary antibodies mentioned above (final dilution of anti-GFP and anti-NeuN 1:2000 and 1:500, respectively). After washing in PBS, the sections were incubated for one hour at room temperature with a cocktail of Alexa Fluor 488 anti-rabbit and Alexa Fluor 568 anti-mouse secondary antibodies (Molecular Probes-Invitrogen, Paisley, UK) with a final dilution of both antibodies of 1:100. The stained sections were washed in PBS and in distilled water and mounted with a water soluble medium suitable for fluorescence microscopy.

The sections were analysed using a confocal laser scanning fluorescence microscope with appropriate filter settings (Axiovision System, Zeiss, Vienna, Austria). First and last sections with EGFP-positive cells (including neurons, glial cells, and other transduced cells if present) and EGFP-positive neurons as well as the position of the injection canal were recorded for each brain. The rostro-caudal spread of immunopositive cells and neurons was calculated using these section numbers and the section thickness of 18 μm.

To assess a potential inflammatory reaction in the brains, two sections per brain in the immediate vicinity of the injection canal were immunostained using an anti-GFAP antibody (polyclonal rabbit anti-Glial Fibrillary Acidic Protein, 1:1000, DakoCytomation, Glostrup, Denmark) and an anti-IBA1 antibody (polyclonal rabbit anti-Iba1, 1:2000, Wako Chemicals, Neuss, Germany) to visualise astrocytes and microglial cells, respectively. Selected sections from brains showing mononuclear infiltrations near the injection canal were additionally stained using an anti-CD3 antibody (monoclonal rabbit anti-CD3, clone SP7, 1:300, Thermo Fisher Scientific, Fremont, CA, USA) to detect T lymphocytes. The sections were deparaffinised and permeabilised as described above. Before blocking of unspecific binding, endogenous peroxidase activity was blocked with 0.6% H_2_O_2_ in methanol for 15 min at room temperature. For IBA1- and CD3 staining, antigen retrieval by heating in 0.01 M citrate buffer (pH 6) for 30 min was necessary. The sections were incubated with the primary antibodies overnight at 4 °C. The immunoreaction was detected using the BrightVision Poly HRP-anti-rabbit kit (ImmunoLogic, Duiven, The Netherlands) according to the instructions of the producer and visualized with diaminobenzidine (Sigma-Aldrich, Vienna, Austria) in 0.03% H_2_O_2_ in PBS. The sections were counterstained with Mayer’s haematoxylin, dehydrated, mounted with a medium soluble in xylene, and analysed by light microscopy.

## Stereological assessment of the number of EGFP-expressing neurons per brain

The number of EGFP-positive neurons within the brain was estimated stereologically using the optical fractionator technique, which is the gold standard for quantification of total numbers of microscopic objects (West et al. 1991; West 1999).

The fractionator is a combination of systematic uniform random sampling and the disector counting principle (Sterio 1984). The countable event was defined as colocalisation of positive immunoreaction against EGFP and NeuN within nucleus or perikaryon of the same cell within a disector probe with the dimensions 210.9 × 210.9 × 16.0 µm (Online Resource 1). The EGFP-positive neurons were counted in a known fraction of the whole brains, with every 20th histological section of the brain selected in a systematic uniform random manner (i.e., sections 20, 40, 60, etc). Thus, the section sampling fraction (ssf) was 1/20. In every section, the profile of the brain was scanned with an oil-immersion objective 40× on a laser scanning microscope as described in the following. The area of the counting frame of the disector, a(frame), was known relative to the area associated with each *x, y* movement of the microscope stage a(*x, y*-step); thus, the area sampling fraction asf was then a(frame)/a(*x, y* step) = 0.14183294. The height h of the disector was 16 μm and the thickness of the histological sections was 18 µm; therefore, the *t*/*h* ratio (Eq. 1) was 1.125 (for illustration, see Online Resource 2). The estimate of the total number of EGFP-positive neurons estN was calculated according to the Eq. 1:

1 $$estN=\sum\limits_{i=1}^n {Q_i^ - } \cdot \frac{t}{h} \cdot \frac{1}{{{\text{asf}}}} \cdot \frac{1}{{{\text{ssf}}}}.$$

The variation in number of neurons in the systematically sampled sections (sampling error) was assessed with use of the coefficient of error CE = (standard error mean)/mean. It was calculated with the quadratic approximation formula of Matheron, modified for use in a stereological context (Gundersen and Jensen 1987; Slomianka and West 2005; West et al. 1991; West 1999). A pilot study performed in one brain proved that the CE for the current section sampling fraction ssf = 1/20 is comparable with the results based on section sampling fraction ssf = 1/10.

**Fig. 1:**
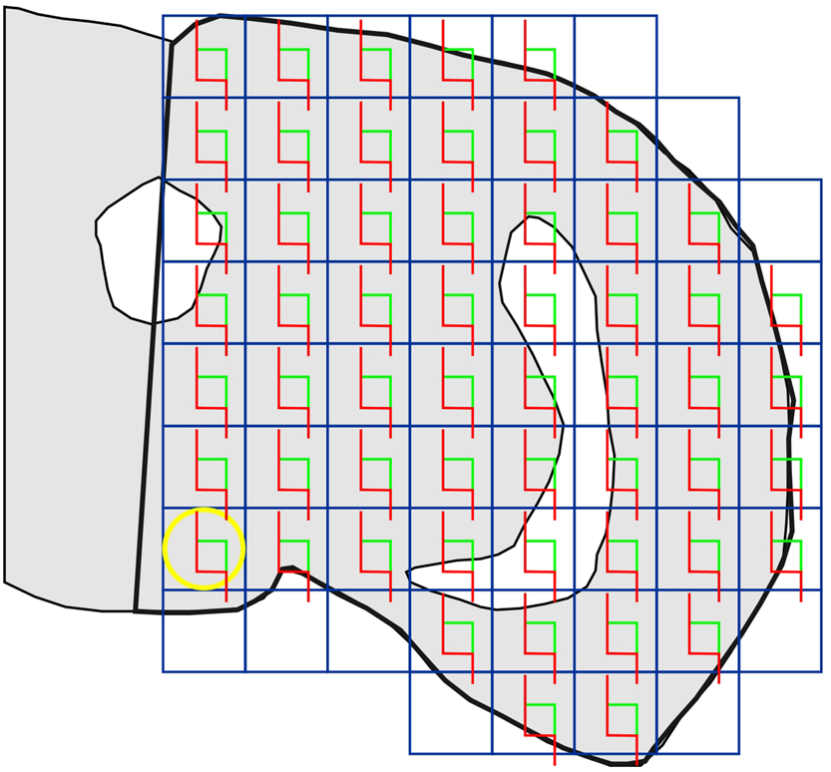
Sampling scheme of a histological brain section with proportionally scaled ocular image fields from confocal microscope (objective 40×). The histological slide was moved in a raster pattern, starting from the ventromedial corner of the profile of the right half of the brain. The *yellow circle* represents the ocular image field with a diameter of 560 μm, and the blue square circumscribing this image field represents the regular predetermined *x, y* step. The area associated with each step of the ocular image field was a(*x, y* step) = 313,600 μm^2^. The number of sampled ocular image fields was recorded. From each field of view containing EGFP-positive neurons, a confocal stack of micrographs was recorded (11 optical sections, inter-section-interval 2 μm, stack height 20 µm). Counting frames of the optical disectors (*red* and *green* borders for forbidden and allowed *lines*, respectively) were superimposed on the stacks of optical sections. The area of the counting frames was a(frame) = 44,478.81 µm^2^. *Grey* = scheme of the right part of a histological section of the brain with ventricles (*white*), *thick black* contours = region of interest

For photodocumentation, the Zeiss LSM 510 Meta confocal microscope (Carl Zeiss Jena GmbH, Jena, Germany) was used to generate registered image stacks for the optical disector method. From all stained histological sections of the brains, microphotographs were taken as follows: the midline of the histological brain section was found and the right half of the brain identified. Starting from the ventromedial corner of the right brain half, image field after image field was evaluated in a raster pattern at an objective magnification of 40×. The microscopic stage was moved in a regular predetermined x,y-step, so that image fields did not overlap, but were joined to each other exactly (Fig. 1). To choose a suitable *x, y* interval, we took into account the recommendations of Harding et al. (1994).

Stereological analysis was performed using the software Ellipse3D (ViDiTo, Košice, Slovakia). The observer (ZT) was blinded to the biological state of the sample.

From each field of view containing any GFP-positive neurons, a confocal stack of micrographs containing 11 optical sections with an inter-section-interval of 2 µm (stack height 20 µm) was recorded.

If EGFP-positive neurons were present also in the left half of the brain, accordingly, image stacks were recorded in the same way after finishing the analysis of the right half of the brain.

### Statistics

Nonparametric statistics was used for data analysis. The Kruskal–Wallis ANOVA and the Mann–Whitney *U* test served to assess the differences in numbers of EGFP-positive neurons per brain and in the rostro-caudal spread of transduced neurons and transduced cells (including neurons, glia, and other cells if applicable) among the groups of animals infected with different types of lentiviral vectors and in dependence of the accessory proteins Vpx or Vpr. The correlation between the quantitative parameters was evaluated using the Spearman correlation coefficient. These tests were used as available in the Statistica Base 11 package (StatSoft, Inc., Tulsa, OK, USA).

The following hypotheses have been tested:

- H0(A) Absolute numbers of EGFP-expressing neurons per mouse brain do not differ after injection of the different virus vectors under study.
- H0(B) The rostro-caudal spread of EGFP-expressing neurons and of all EGFP-expressing cells in the mouse brain does not differ after injection of the different virus vectors under study.
- H0(C) The accessory protein Vpx/Vpr does not influence the absolute numbers of EGFP-expressing neurons in the mouse brain after injection of the respective virus vectors.
- H0(D) The accessory protein Vpx/Vpr does not influence the rostro-caudal spread of EGFP-expressing neurons nor of all EGFP-expressing cells in the mouse brain after injection of the respective virus vectors.
- H0(E) Absolute numbers of EGFP-expressing neurons per mouse brain after injection of the transgenic virus vectors do not correlate with the rostro-caudal spread of EGFP-expressing neurons nor of EGFP-expressing cells.

## Results

### Infection of primary mesencephalic cell cultures

Infectivity of the vectors was tested on mouse primary mesencephalic cell cultures. The transduction potential of all viruses has been confirmed for both neurons and glial cells by positive double immunofluorescence for EGFP and NeuN and EGFP and GFAP, respectively (Online Resource 3). The transduction efficiency for neurons ranged between 20% for PBjSEW(Vpx) and 37% for PBjSEW and HIV-2SEW (Online Resource 4).

### Transduction of brain cells in vivo and distribution of positive cells along anatomical structures

**Fig. 2:**
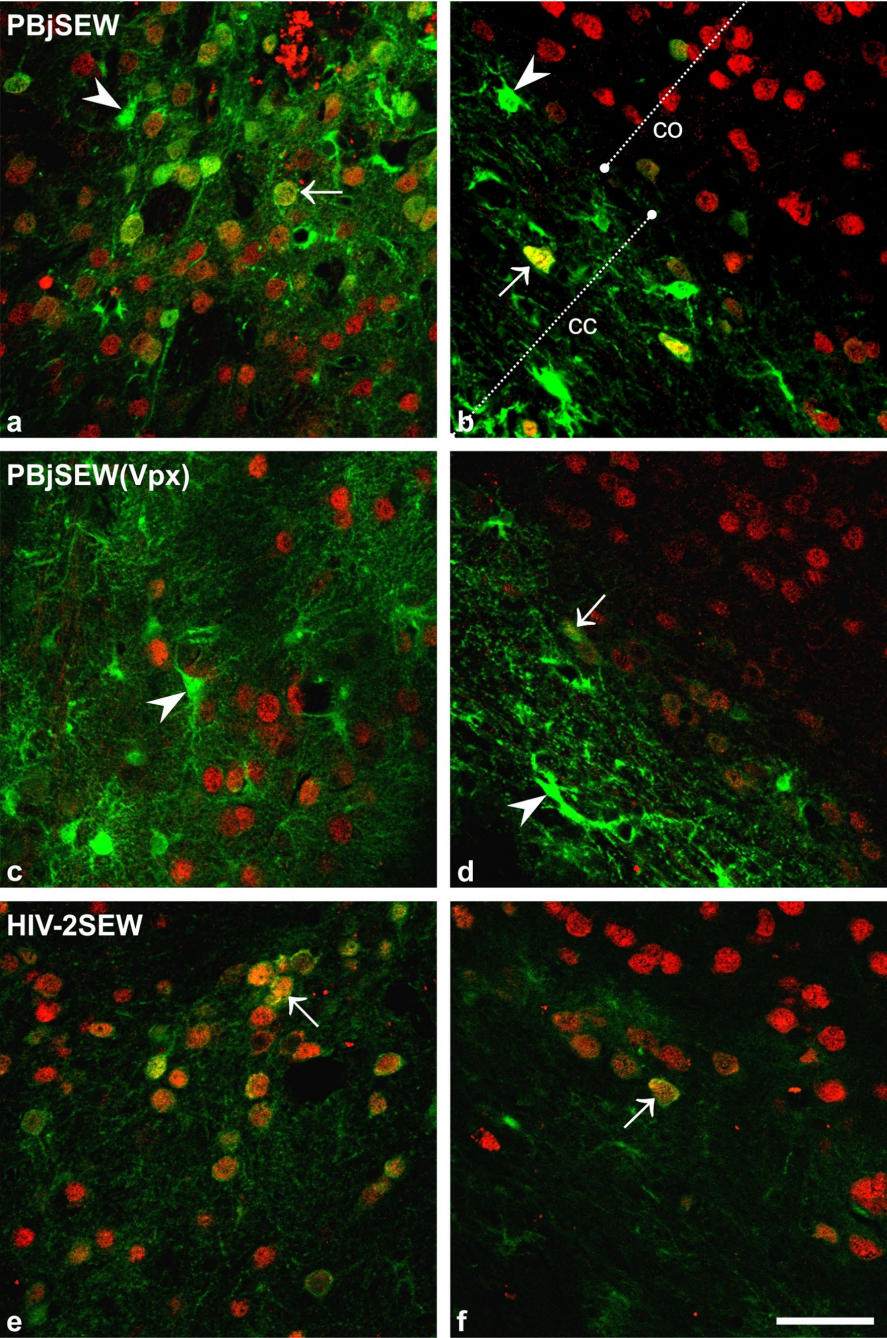

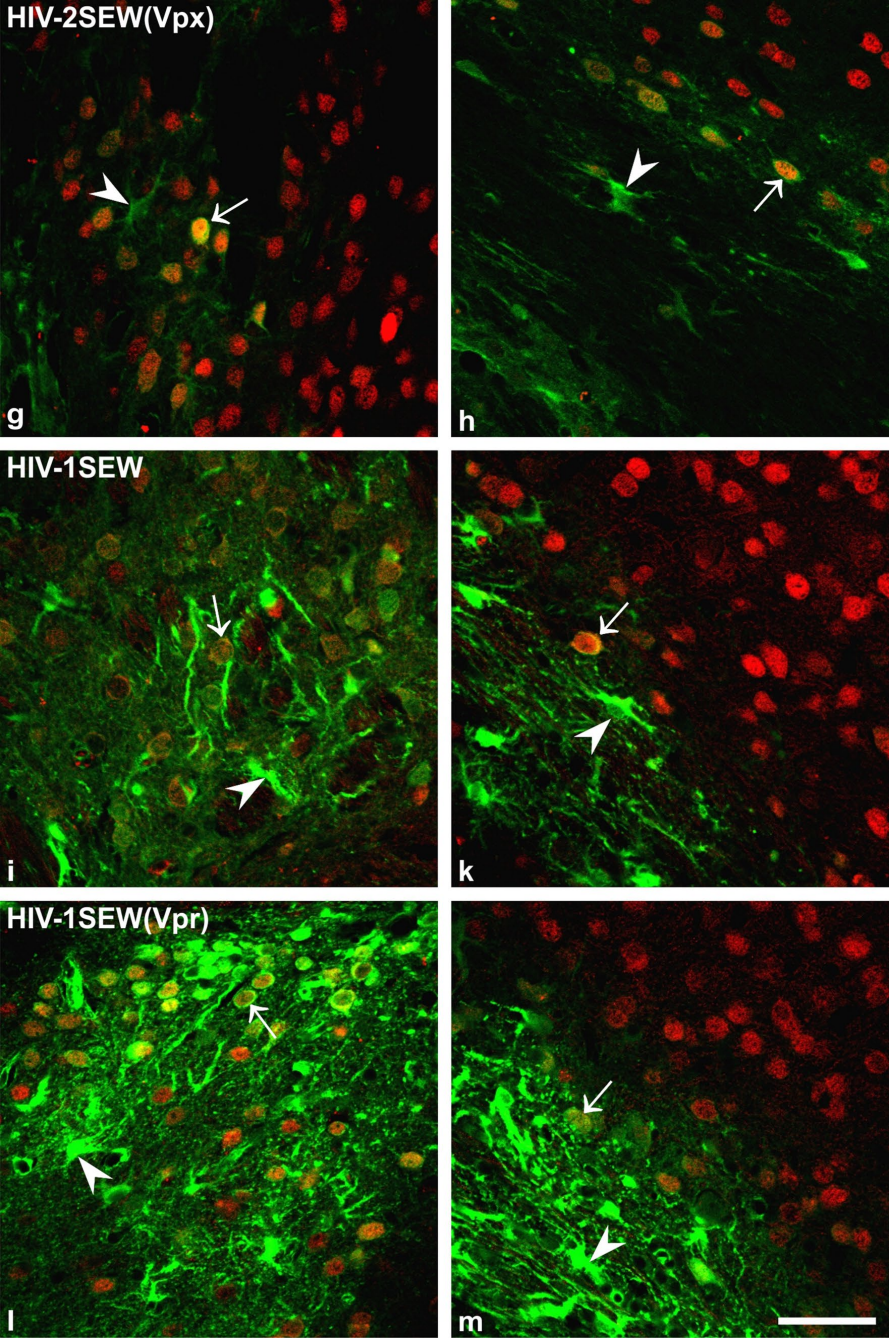
Transduction of neurons and glial cells of the mouse brain by lentiviral vectors-qualitative comparison. Micrographs **a, c, e, g, i**, and **l** have been taken from location 2 marked in Fig. 3, micrographs **b, d, f, h, k**, and **m** from location 1. Note that EGFP expression (*green* staining) was always stronger in glial cells (*arrowheads*) than in neurons (*arrows*), but that proportion of transduced neurons and glial cells differed according to the vector that was injected into the brain. Vectors with the accessory proteins Vpx or Vpr seemed to evoke increased EGFP expression and thus higher intensity of the fluorescence signal compared to the same vector without this protein. In the PBjSEW vector, the Vpx protein additionally changed the preferred infection site from neurons to glial cells (compare** a** and** b** vs.** c** and** d**). Double fluorescence immunohistochemistry, EGFP = *green*, NeuN (neuronal marker) = *red*, overlay = *yellow*; thickness of the optical section = 2 µm; co = cortex, cc = corpus callosum, *scalebar *50 µm

**Fig. 3:**
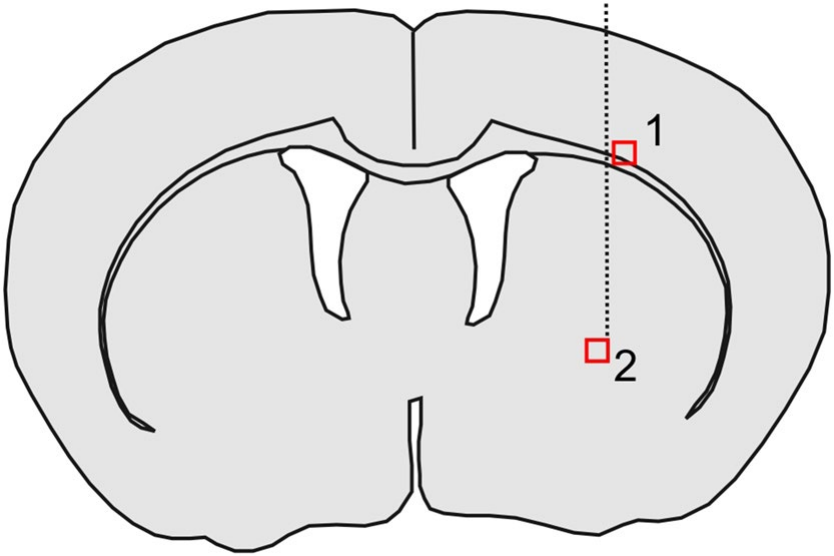
Image fields used for qualitative comparison of neuronal and glial transduction by transgenic viruses. Scheme of a coronal section of a mouse brain according to Paxinos and Franklin (2003) at the level of the injection canal (*dotted line*). *1* Image field including cortex and corpus callosum; *2* image field at the ventral end of the injection canal, ventral part of the corpus striatum

Transduction of cells of the mouse brain after injection of EGFP-harbouring lentiviral vectors was analysed in exhaustive serial histological sections after double immunofluorescence staining for EGFP and NeuN. Positive EGFP immunostaining could be found in all brains under study, in the cytoplasm of neurons as well as of glial cells including ependyma. The fluorescence signal of glial cells was always distinctly stronger than that of neurons within the same section (Fig. 2; see Fig. 3 for definition of the photographed brain sites).

Neurons stained reliably with the NeuN antibody, as could be confirmed by cell morphology and position of the positive cells. Positive immunoreaction could be found in the nucleus, the cytoplasm, or nucleus and cytoplasm of the neurons. In some cases, NeuN bound also to nucleus and/or cytoplasm of single ependymal cells lining the lateral and the third ventricles, but never to ependymal cells covering the choroidea. Due to the specific shape and position of the ependymal cells, they could be easily distinguished from neurons in spite of their occasionally positive NeuN staining.

Neurons positive for EGFP could be readily detected as cells whose cytoplasm and nuclei showed green and red fluorescence, respectively (anti-GFP/Alexa Fluor 488 Anti-Rabbit, anti-NeuN/ Alexa Fluor 568 Anti-Mouse), or as cells with yellow overlay fluorescence of the cytoplasm (Fig. 2). These cells were defined as a countable event for quantification of transduced neurons.

Immediately adjacent to the injection canal single slightly EGFP-positive macrophages could be found irrespective of the injected vector. The injection canal itself was still identifiable after 3 weeks observation time as a slender band with slightly altered tissue structure without any opening in the middle. Microglial reaction was least marked for the HIV-2SEW-based vectors. In these samples, nearly, all detected microglial cells showed a ramified phenotype, and their number was only slightly increased around the injection canal in comparison with the contralateral side. After injection of the HIV-1SEW and PBjSEW-based vectors, microglial reaction on the injection site was more pronounced than for HIV-2SEW vectors, including visibly increased numbers of IBA1-positive cells and a larger number of cells showing a bushy or rounded phenotype around the injection canal. Rounded microglial cells corresponded to the EGFP-positive macrophages described above. In addition, HIV-1SEW-based vectors evoked a particularly strong microglial reaction around the ventricles, which was considerably less pronounced with the PBjSEW vectors and nearly absent with HIV-2SEW vectors. For all vectors, astrocytes on the ipsilateral brain side showed a markedly increased GFAP expression, whereas contralaterally, they were only slightly immunopositive or negative. This left–right asymmetry of GFAP-immunoreactivity was least pronounced for the HIV-2SEW-derived vectors (Online Resource 5). Small numbers of T lymphocytes were found scattered in the vicinity of the injection canal. Moreover, they were a part of small clusters of mononuclear cells, mostly in form of perivascular cuffs, found on the ipsilateral side of the brain. These mononuclear cell clusters were most prominent in samples injected with the HIV-1SEW-derived vectors (not shown). The accessory proteins Vpx/Vpr had no visible effect on glial reaction and T-cell infiltration when the results of vectors derived from the same parental virus were compared (Online Resource 5).

**Fig. 4:**
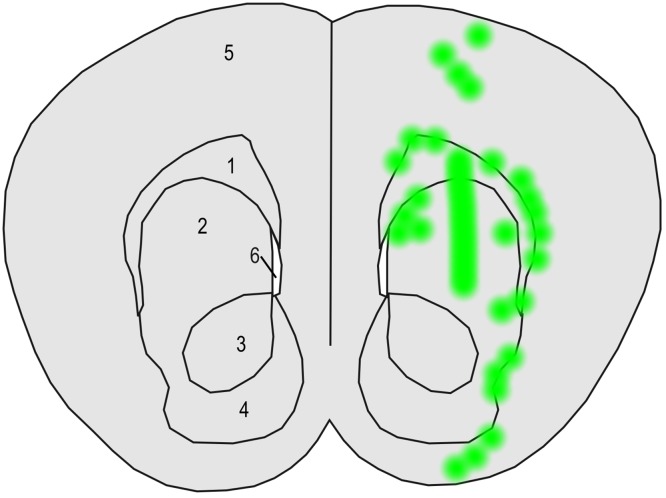
Schematic representation indicating the distribution of EGFP-positive cells (*green* regions) within and around the corpus striatum-example. Coronal section of a mouse brain according to Paxinos and Franklin (2003) at the level of the anterior end of the lateral ventricles. *1* Corpus callosum; *2* nucleus caudatus and putamen; *3, 4* core and shell of nucleus accumbens; *5* cortex telencephali; *6* lateral ventricle. EGFP-positive neurons and glial cells were spread along the injection canal which runs from parietal cortex to corpus striatum, as well as along the corpus callosum and in groups within the corpus striatum itself

The distribution of EGFP immunopositivity depended on virus and infected cell type, and was influenced by brain anatomy (Fig. 4). EGFP-positive cells were mostly constrained to the right half of the brain. Only for the HIV-1SEW and HIV-2SEW(Vpx) viruses, single immunopositive neurons and glial cells could be found in the left brain half near the midline in vicinity to the corpus callosum. In addition, PBjSEW(Vpx) and HIV-1SEW(Vpr) transduced single glial cells, but no neurons were detected in the left half of the brain.

Distribution of EGFP-positive cells in relation to brain structures was comparable in all animals under study, regardless of the injected virus type. Positive cells surrounded the injection canal, i.e., they were found within the dorsal regions of the telencephalic cortex and in the corpus striatum. Discrete clouds of immunopositive cells could also be detected within a certain distance of the injection canal in nucleus caudatus, putamen, and accumbens nucleus and spreading along the corpus callosum and its radiatio, preferentially at the borderline between corpus callosum and striatum (Fig. 4).

In all brains injected with the HIV-1SEW vector and in single brains injected with PBjSEW, PBjSEW(Vpx), HIV-2SEW(Vpx), and HIV-1SEW(Vpr), the injected viruses spread rostrally towards the bulbus olfactorius. As a result, single EGFP-positive neurons could be found in the olfactory bulb. Injection of the HIV-2SEW, HIV-1SEW, and HIV-1SEW(Vpr) vectors resulted in some positive glial cells but not neurons in the olfactory bulb. In contrast, the rostral part of the hemispheres remained negative, and the main portion of positive neurons followed as described above and in Fig. 4. Caudalmost EGFP-positive neurons were identified in the cerebellar stratum granulosum and the hippocampus after transduction by the HIV-1SEW vector.

All vectors except PBjSEW transduced ependymal cells lining lateral and third ventricles. If the fluorescence signal of ependymal cells lining the third ventricle was strong, positive ependymal cells could also be found in the left half of the third ventricle (HIV-1SEW, HIV-1SEW(Vpr), PBjSEW(Vpx)) and in single cases with HIV-1SEW injection even in the left lateral ventricle.

### Preferential in vivo infection of glial cells and neurons depending on virus type and presence of accessory proteins

Although all viruses infected neurons as well as glial cells and evoked EGFP expression in both cell types, the preference of each of the viruses and the EGFP expression levels differed (Table 1). The HIV-2SEW vector was the most specific one for neurons of all tested viruses.

**Table 1:** Preferentially infected cell type, amount of transduced cells and EGFP expression levels as estimated by qualitative microscopic analysis

Virus	Preferred cell type	Amount of transduced cells and EGFP expression levels	
		Neurons	Glial cells
PBjSEW	Neurons	Many, middle intensity	Intermediate, high intensity
PBjSEW (Vpx)	Glial cells	Very few, low intensity	Many, high intensity
HIV-2SEW	Neurons	Intermediate, middle intensity	Very few, middle intensity
HIV-2SEW (Vpx)	Neurons, also glial cells	Intermediate, middle intensity	Intermediate, high intensity
HIV-1SEW	Glial cells	Intermediate, middle intensity	Many, high intensity
HIV-1SEW (Vpr)	Glial cells	Intermediate, middle to high intensity	Many, very high intensity

To illustrate the different cell preference of the viruses, micrographs of two standard brain parts (Fig. 3) per virus are shown for comparison (Fig. 2).

### Numbers of EGFP expressing neurons per brain

**Fig. 5:**
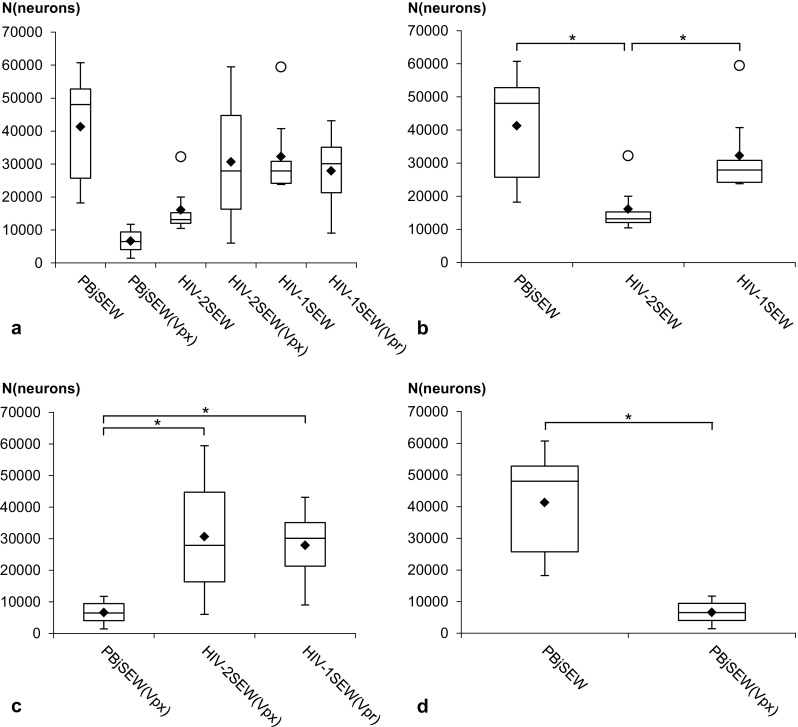
Numbers of EGFP-positive neurons per brain (N(neurons)) after intracerebral injection of PBjSEW, HIV-2SEW, and HIV-1SEW lentiviral vectors carrying the EGFP reporter gene with or without the accessory proteins Vpx/Vpr. **a** Overview of all brains under study. **b** Numbers of transduced neurons were larger after injection of PBjSEW and HIV-1SEW when compared to HIV-2SEW. **c** Numbers of transduced neurons were larger after injection of HIV-2SEW(Vpx) and HIV-1SEW(Vpr) when compared to PBjSEW(Vpx). **d** Accessory protein Vpx/Vpr caused significant decrease of neuronal transduction rates for vector PBjSEW but not for the other vectors. Only differences significant at *p* < 0.05 (*) are shown. *Boxes* span the interquartile range of data on the *y*-axis. The inside *lines* indicate the median and the *black diamonds* mean values. *Whiskers* extend to the minimum and maximum values excluding outliers (*white circles*)

The absolute numbers of transduced, i.e., infected and EGFP expressing neurons within the whole mouse brains as assessed by stereology, ranged between 1428 (PBjSEW(Vpx)) and 60,758 (PBjSEW) with a mean of 25,831 neurons for all vectors (Fig. 5a, Online Resource 6). Numbers of transduced neurons differed depending on the parental viruses (SIVsmmPBj, HIV-2, and HIV-1) of the vectors (Fig. 5b, c). Only for vector PBjSEW, a difference in transduction efficiency of neurons depending on the presence of the Vpx accessory protein could be detected (Fig. 5d).

**Fig. 6:**
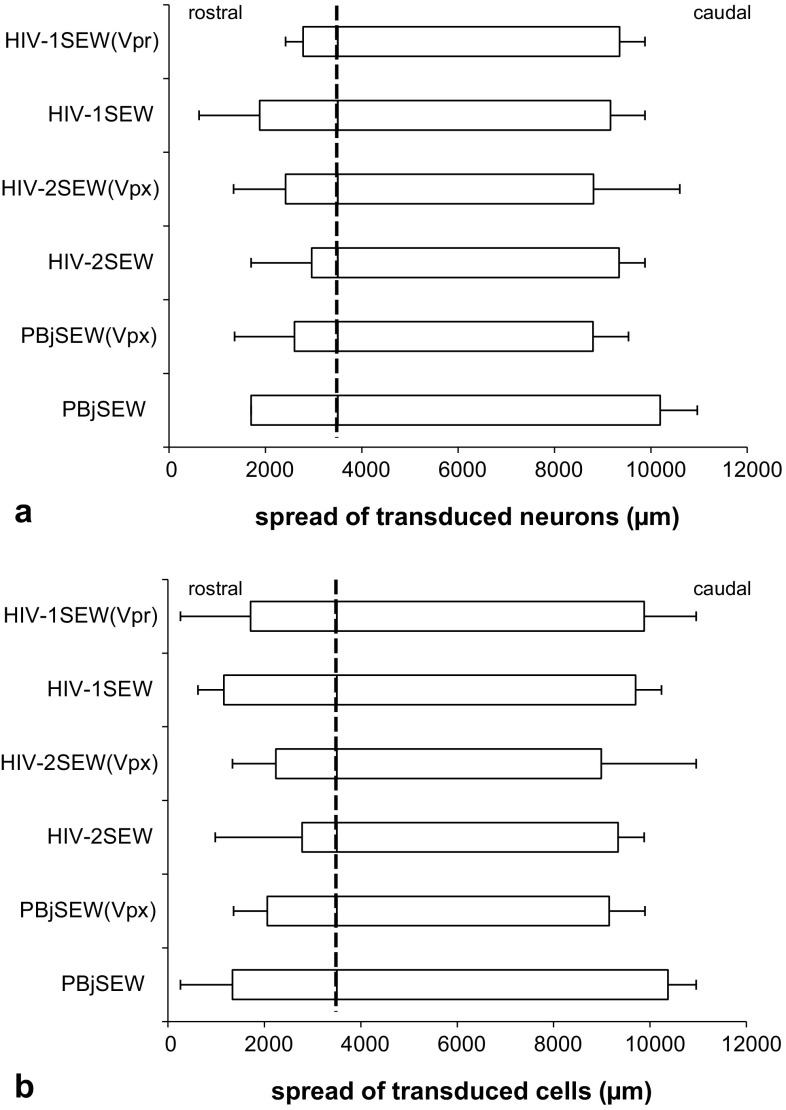
Rostro-caudal spread of EGFP-positive neurons (**a**) and brain cells including neurons, glia, and other cells if applicable (**b**); within the mouse brain after intracerebral injection of PBjSEW, HIV-2SEW, and HIV-1SEW lentiviral vectors carrying the EGFP reporter gene with or without the accessory proteins Vpx/Vpr. The brains were superimposed in a virtual coordinate system so that the injection canal (*dashed line*) is displayed in one plane. The boxes depict median values of six brains per vector, the whiskers maximal spread in rostral and caudal directions. Note that for all vectors, transduced neurons and cells could be found in a larger distance from the injection canal in caudal direction. No significant differences were observed between vectors or depending on the absence or presence of Vpx/Vpr. The chart does not depict the total amount of transduced cells. If at least one positive cell profile was found in the respective histological section, it was regarded as positive. The part of the rostral brain pole between olfactory bulb and striatum that did not contain transduced cells (see chapter “Transduction of brain cells in vivo and distribution of positive cells along anatomical structures”) is not considered in this graph

Rostralmost EGFP-expressing neurons were detected in a distance of up to 2880 μm rostrally to the injection canal in the bulbus olfactorius (HIV-1SEW), caudalmost positive neurons 3960 μm from the injection canal (PBjSEW) in the caudal pole of the telencephalon, hippocampus, and cerebellum. Maximal spread of all transduced cells was 3240 μm (PBjSEW and HIV-1SEW(Vpr)) in rostral and 3960 μm (PBjSEW, HIV-2SEW(Vpx), and HIV-1SEW(Vpr)) in caudal direction from the injection canal (Fig. 6). There were no significant differences in rostral, caudal, and total spread of transduced neurons or all transduced cells between vectors or depending on the presence of the Vpx or Vpr protein.

### Correlations between quantitative parameters

The non-parametric Spearman rank order correlations between quantitative parameters are listed in Table 2. The numbers of EGFP-positive neurons were moderately or strongly correlated with the spread of positive cells (including neurons, glia, and other cells of the brain) and neurons both rostrally as well as caudally to the injection canal.

**Table 2:** Spearman rank order correlations between the quantitative parameters assessed in mouse brains after injection of lentiviral vectors carrying the EGFP reporter gene. Correlations significant at *p* < 0.05 are marked (*)

	Spread of transduced cells (µm)			Spread of transduced neurons (µm)		
	Total rostro-caudal	Rostrally to the injection canal	Caudally to the injection canal	Total rostro-caudal	Rostrally to the injection canal	Caudally to the injection canal
Number of transduced neurons	0.70*	0.54*	0.73*	0.69*	0.49*	0.64*
Rostro-caudal spread of transduced cells (µm)	–	0.89*	0.88*	0.87*	0.71*	0.78*
Spread of transduced cells rostrally to the injection canal (µm)	–	–	0.59*	0.69*	0.70*	0.55*
Spread of transduced cells caudally to the injection canal (µm)		–	–	0.87*	0.55*	0.87*
Rostro-caudal spread of transduced neurons (µm)		–	–	–	0.77*	0.91*
Spread of transduced neurons rostrally to the injection canal (µm)		–	–	–	–	0.49*

Autocorrelations and repeating values are replaced by the sign

## Discussion

In this work, we have directly compared the transduction potential, infection preferences, and distribution of non-replicating gene transfer vectors derived from sooty mangabey monkey SIV strain PBj, HIV-2, and HIV-1 after unilateral injection into the brain of adult, immunocompetent mice. The effect of accessory protein Vpx/Vpr presence in the vector particles on these parameters was analysed as well.

The infectivity of all vectors under study and their ability to transduce brain cells has been confirmed in vitro. Hitherto, numerous studies have shown the efficacy of HIV-derived vectors in gene transfer into non-dividing neurons including primary cerebral cortical neurons, pyramidal neurons, and neuronal progenitor cells (D´Costa et al. 2003; Wong et al. 2006; Zhang et al. 2006; Piersanti et al. 2013). To date, even though PBj-derived vectors were efficient in infection of human quiescent primary monocytes and dendritic cells as well as G_0_-arrested human cell lines, the capability to infect neurons has not been described yet (Mühlebach et al. 2005; Wolfrum et al. 2007; Grabski et al. 2011). In our studies, in-vitro EGFP expression was detected in both neuronal and glial cells, regardless of the vector used. We were able to show that not only HIV, but also PBj-derived vectors are suitable for infection of neurons (NeuN+), i.e., the main target cells of research in this field. To our best knowledge, this is the first evidence of neuronal transduction using vectors derived from the SIVsmm PBj strain.

In a next step, we have carefully compared the in vivo performance of these lentivirus vectors in the mouse brain. In line with the in vitro observation, EGFP expression was detected in both neuronal and glial cells, irrespective of the vector origin (PBj, HIV-2, HIV-1) and absence or presence of the accessory protein Vpx/Vpr. This is in contrast to a number of other studies using VSV-G-pseudotyped HIV-based lentiviral vectors in murine or rat brain, in which a clear prevalence for transduced neurons was observed (e.g., Naldini et al. 1996a; Blömer et al. 1997; Bensadoun et al. 2000). However, it has been demonstrated that beside the observed transduction specificity, the choice of the promotor sequence can invoke exclusive expression of the gene of interest in either glial cells or (specific) neurons (Jakobsson et al. 2003; Delzor et al. 2013; Schulze et al. 2015). To avoid this promoter-driven restriction of EGFP expression, we used the strong, ubiquitous SFFV promoter with described activity in neuronal cells (Bender et al. 2007). From qualitative microscopic evaluation, it seems that EGFP expression levels were visibly higher in glial cells than in neurons (cf. Fig. 2), which would be in agreement with the EGFP expression data of HIV-1 vectors published previously (Baekelandt et al. 2002). Diversity in the expression levels might be accounted to differences in promoter activity between these two cell types and/or to disparity in transcription and translation rate among these cells (Stoykova et al. 1985; Delzor et al. 2013).

In our in vivo experiments, injection canals were still detectable after an observation period of 3 weeks. Single EGFP-processing macrophages were observed around the needle tract with an additional slight lymphocytic infiltration for all vectors. Activation of microglial cells was more prominent after injection of HIV-1SEW and PBjSEW-based vectors. In general, former studies have confirmed a low pathogenicity of lentiviral vectors (Naldini et al. 1996a; Blömer et al. 1996; Baekelandt et al. 2002; Sinnayah et al. 2002; Wong et al. 2006). Blömer et al. (1997) described a slight infiltration of immune cells after intracerebral injection of different viral vectors as well as after saline control injection and interpreted it as a reaction to the trauma that disappears after a survival time of 6 weeks. The traces of the needle tract with single EGFP-containing macrophages and activated microglia in our experiment might represent an intermediate healing stage of the puncture rather than a reaction to the virus, similarly as suggested by Baekelandt et al. (2002). However, visible reaction differences between the viral vectors were observed. To distinguish between a reaction of the brain to insertion of the needle and application of a certain volume and a reaction to the virus itself, additional studies employing sham injections of a non-immunogenic liquid would be necessary. Similarly, increased GFAP expression in astrocytes, as found on the ipsilateral brain side in our study, is described to be an important component of the reaction of the brain to a number of different injuries or infections (e.g., Eng and Ghirnikar 1994; Johnson et al. 1995) and thus cannot be attributed exclusively to the brain trauma or the effects of the viral vectors without further comprehensive studies addressing reactive changes in the whole brain.

To our knowledge, the distribution of transduced cells within the whole brain after intracerebral injection of lentiviral vectors has not been systematically described yet. Most authors concentrate on a specific target structure such as the nigrostriatum or specific nuclei (Naldini et al. 1996a; Blömer et al. 1997; Bensadoun et al. 2000; Sinnayah et al. 2002). Baekelandt et al. (2002) reported a similar distribution of transduced cells along the corpus callosum after intrastriatal vector injection as found in our study. In contrast to Kato et al. (2011) who studied the extent of retrograde transport of lentiviral vectors pseudotyped with chimeric rabies/VSV-G fusion envelope after intrastriatal injection and found transduced neurons in upstream regions of the striatum (primary motor and somatosensory cortex, parafascicular thalamic nucleus, and substantia nigra pars compacta), we did not detect EGFP-expressing neurons in other cortical regions than surrounding the injection canal. This transduced cell population might rather be attributed to spillage during removal of the needle than to vector particle transport.

Interestingly, we found EGFP-positive neuronal cell bodies as far from the injection site as the olfactory bulb-regularly with the HIV-1SEW vector and sometimes with all the other vectors except HIV-2SEW. A plausible way of spread might be the retrograde way along the olfactory system from piriform lobe (where vector particles might arrive even by diffusion from the injection site) via olfactory tracts to olfactory bulb. It seems that the vector particle is transported rather than the gene product, since immunopositivity was restricted to the somata of olfactory bulb neurons and the immediately adjacent parts of axons and dendrites. No EGFP could be detected in the olfactory tract, and it has to be taken into account that a minimum of two synapses has to be bridged on the way from piriform lobe to olfactory bulb. The mechanism of the retrograde transport of viral particles within the brain is not known, but glycoproteins of the viral envelope seem to play a role during the interaction with host cells and endosomal vector transport (Desmaris et al. 2001; Kato et al. 2011; Carpentier et al. 2012; Schoderboeck et al. 2015). Our results confirm that transduction of olfactory bulb neurons is possible for simple VSVG-pseudotyped lentiviruses, even if rabies or chimeric rabies/VSVG pseudotyping might enhance retrograde axonal transport of vectors (Kato et al. 2011; Carpentier et al. 2012; Schoderboeck et al. 2015). Our results provide also evidence for anterograde transport of the injected vector particles, since transduced cell somata could be found in cerebellum and hippocampus. At least for the cerebellum, a simple transport of the reporter gene product along the axon (Baekelandt et al. 2002; Jakobsson et al. 2003) can be excluded as an explanation, since multiple synapses would have to be crossed. The general possibility of transport of VSVG-pseudotyped HIV-1 viral vectors in conducting direction (from dendrites to cell body) has been shown by Desmaris et al. (2001). Glial cells, particularly astrocytes, might contribute to retro- and anterograde transport of the viral vectors, since distribution of transduced glial cells coincides with that of neurons.

From our results, it seems that the midline of the brain represents a considerable barrier to viral spread, which is also supported by Baekelandt et al. (2002). However, according to our results, highly infective viruses (HIV-1SEW, HIV-1SEW(Vpr), PBjSEW(Vpr)) are not only able to enter ependymal cells of the ipsilateral lateral ventricle, but they seem to cross these cells, and reach the contralateral site of the third and via foramen interventriculare even the contralateral lateral ventricle by liquor (HIV-1SEW). Here, they also infected ependymal cells but did not enter the nervous tissue, a finding that was supported by our own observations after injection of the vector particles into the lateral ventricles (data not shown; see also Baekelandt et al. 2002; Porlan et al. 2016). Since especially for both HIV-1SEW derived vectors, a marked accumulation of microglial cells around the ventricles could be observed, and a certain contribution of these cells to vector transport into the liquor cannot be excluded.

Tropism of lentiviral vectors to neurons vs. glial cells has been described to depend on their pseudotyping, their transcriptional and post-transcriptional elements used to control transgene expression, the animal species used as a model organism and their developmental stage, the timepoint of observation, the brain areas targeted, and the intracerebral delivery protocol (reviewed by Delzor et al. 2013). In our experiment, most of these parameters were kept constant including the internal organization of the transfer vectors. The viruses under study only differed regarding the parental viruses and the absence or presence of the accessory proteins Vpx or Vpr. However, they differed considerably with respect to the cell type they preferentially infected, HIV-2SEW being the most specific for neurons (Table 1), although its transduction efficiency was lower than for the other vectors (Fig. 5b). Interestingly, HIV-2SEW and HIV-2SEW(Vpx) evoked also the lowest activation of astrocytes and microglial cells in comparison with all other vectors under study (Online Resource 5), suggesting that a low inflammatory and reactive response of the brain might actually enhance the specific infection of neurons, whereas increased inflammation seems to support the overall transduction efficiency. This effect appears to be associated with the type of the parental virus, since the presence of the accessory Vpx/Vpr proteins did not influence the reaction of the nerve tissue considerably. However, further experiments searching for correlations between inflammatory changes in the complete brain and in vivo transduction efficiency would have to be carried out to clarify these relationships.

The absolute numbers of transduced neurons within the mouse brain differed among the different viruses tested in our study, although their general composition, i.e., membrane of host cells used for particle production, virus surface protein, and the design of the transfer vector, were similar. Vector HIV-2SEW yielded the significantly lowest infection rates. It was, however, the most specific for neurons. Among the vectors carrying the Vpx/Vpr accessory proteins, PBjSEW(Vpx) generated the lowest numbers of transduced neurons, seemingly due to a preference for glial cell transduction that could be clearly observed by simple fluorescent microscopy (cf. Fig. 2a–d). The immediate cause of the different vector performances is not known.

The Vpx/Vpr accessory proteins were originally expected to increase numbers of transduced neurons, since they are described to be indispensable for infection of quiescent/G_0_ cells (Mangeot et al. 2002; Wolfrum et al. 2007; Srivastava et al. 2008; Mashiba and Collins 2012) and to increase expression levels of HIV vector genes (Dupuy et al. 2005; Goujon et al. 2007). In our experiment, no significant impact of the Vpx or Vpr protein on the number of transduced neurons could be detected with the exception of PBjSEW vectors as described above. A possible increase of EGFP expression intensity due to Vpx or Vpr presence would have to be quantified and compared using appropriate methods, e.g., in vitro quantification of EGFP-protein contents within the whole brain.

The rostro-caudal spread of transduced cells found in our experiment was similar to that found by Kato et al. (2011) but considerably larger than that mostly given in literature (e.g., Naldini et al. 1996a; Blömer et al. 1997; Bensadoun et al. 2000; Baekelandt et al. 2002; Sinnayah et al. 2002), probably because most authors concentrated in their studies on a specific region of interest and used detection systems for the reporter gene that are less sensitive than fluorescence immunohistochemistry. In addition, slightly higher volume and amount of virus particles in our study might have contributed to this increased dissemination of transduced cells, even if retro- and anterograde vector transport is not taken into account. Neither rostro-caudal spread, nor rostral or caudal spread of EGFP-positive cells from the injection site differed between the vectors. However, the number of transduced neurons was correlated with spread distances. Viral vector particles that are able to disseminate well within the brain thus seem to be able to infect a larger number of neurons. This cannot be explained simply by the fact that spreading vectors are able to reach more neurons or brain cells, since rostral- and caudalmost transduced cells were very few and did not contribute considerably to the assessed cell numbers. It might be assumed that vectors able to enter cells efficiently are also more easily transported in retro- and anterograde directions.

Numbers of transduced glial cells have not been assessed in our study, since an unequivocal definition of the countable event was not possible even after double staining with GFAP (data not shown). Individual cell somata within the optical disector could not be identified in the dense tangle of intensively EGFP-expressing cell processes (Fig. 2).

We conclude from our findings that after intracerebral injection of lentiviral vectors with the same transfer vector design and vector particle pseudotype, but from different parental viruses, (1) distribution of transduced cells did not differ significantly among the tested lentiviral vectors and depended rather on the (micro-)anatomical structure of the brain. (2) Different tropism of the viruses for neurons and glial cells could be detected, the most specific for neurons being vector HIV-2SEW. However, all vectors under study transduced glial cells as well as neurons. (3) PBjSEW and HIV-1SEW transduced more neurons per brain than HIV-2SEW, whereas in the presence of the accessory proteins Vpx or Vpr, HIV-2SEW(Vpx) and HIV-1SEW(Vpr) showed the highest transduction efficiencies. The distance of spread of transduced cells from the injection canal did not differ among the viruses but correlated with the number of EGFP-expressing neurons. These could be found as far from the injection site as bulbus olfactorius, hippocampus, and cerebellum, even if vectors employing the VSV-G envelope glycoprotein are described to be transported only a short way (Baekelandt et al. 2002; Kato et al. 2011). (4) The presence of the accessory proteins Vpx or Vpr did not result in higher numbers of transduced neurons per brain. For vector PBjSEW, the presence of Vpx significantly decreased the number of transduced neurons per brain, possibly due to a preferred infection of glial cells. Thus, the parental virus seems to considerably influence cellular tropism and transduction efficiency. This should be taken into account in addition to the necessity of precise injection and choice of an appropriate envelope protein for pseudotyping when considering lentiviral vectors for targeted transduction of a defined population of brain cells.

## Electronic supplementary material

Below is the link to the electronic supplementary material.

Supplementary material 1 (JPG 572 KB)

Supplementary material 2 (JPG 187 KB)

Supplementary material 3 (JPG 520 KB)

Supplementary material 4 (JPG 394 KB)

Supplementary material 5 (DOCX 14 KB)

Supplementary material 6 (JPG 5347 KB)

Supplementary material 7 (XLSX 13 KB)

## References

[CR1] Baekelandt V, Claeys A, Eggermont K, Lauwers E, De Strooper B, Nuttin B, Debyser Z (2002). Characterization of lentiviral vector-mediated gene transfer in adult mouse brain. Hum Gene Ther.

[CR2] Belshan M, Mahnke LA, Ratner L (2006). Conserved amino acids of the human immunodeficiency virus type 2 Vpx nuclear localization signal are critical for nuclear targeting of the viral preintegration complex in non-dividing cells. Virology.

[CR3] Bender FLP, Fischer M, Funk N, Orel N, Rethwilm A, Sendtner M (2007). High-efficiency gene transfer into cultured embryonic motoneurons using recombinant lentiviruses. Histochem Cell Biol.

[CR4] Bensadoun JC, Déglon N, Tseng JL, Ridet JL, Zurn AD, Aebischer P (2000). Lentiviral vectors as a gene delivery system in the mouse midbrain: Cellular and behavioral improvements in a 6-OHDA model of Parkinson’s disease using GDNF. Exp Neurol.

[CR5] Blömer U, Naldini L, Verma IM, Trono D, Gage FH (1996). Applications of gene therapy to the CNS. Hum Mol Genet.

[CR6] Blömer U, Naldini L, Kafri T, Trono D, Verma IM, Gage FH (1997). Highly efficient and sustained gene transfer in adult neurons with a lentivirus vector. J Virol.

[CR7] Carpentier DCJ, Vevis K, Trabalza A, Georgiadis C, Ellison SM, Asfahani RI, Mazarakis ND (2012). Enhanced pseudotyping efficiency of HIV-1 lentiviral vectors by a rabies/vesicular stomatitis virus chimeric envelope glycoprotein. Gene Ther.

[CR8] D’Costa J, Harvey-White J, Qasba P, Limaye A, Kaneski CR, Davis-Warren A, Brady RO, Bankiewicz KS, Major EO, Arya SK (2003). HIV-2 derived lentiviral vectors: Gene transfer in Parkinson’s and Fabry disease models in vitro. J Med Virol.

[CR9] Déglon N, Hantraye P (2005). Viral vectors as tools to model and treat neurodegenerative disorders. J Gene Med.

[CR10] Delzor A, Escartin C, Déglon N (2013). Lentiviral vectors: A powerful tool to target astrocytes in vivo. Curr Drug Targets.

[CR11] Demaison C, Parsley K, Brouns G, Scherr M, Battmer K, Kinnon C, Grez M, Thrasher AJ (2002). High-level transduction and gene expression in hematopoietic repopulating cells using a human immunodeficiency virus type 1-based lentiviral vector containing an internal spleen focus forming virus promoter. Hum Gene Ther.

[CR12] Desmaris N, Bosch A, Salaün C, Petit C, Prévost MC, Tordo N, Perrin P, Schwartz O, De Rocquigny H, Heard JM (2001). Production and neurotropism of lentivirus vectors pseudotyped with lyssavirus envelope glycoproteins. Mol Ther.

[CR13] Dupuy FP, Mouly E, Mesel-Lemoine M, Morel C, Abriol J, Cherai M, Baillou C, Nègre D, Cosset FL, Klatzmann D, Lemoine FM (2005). Lentiviral transduction of human hematopoietic cells by HIV-1- and SIV-based vectors containing a bicistronic cassette driven by various internal promoters. J Gene Med.

[CR14] Eleftheriadou I, Mazarakis ND (2015). Lentiviral vectors for gene delivery to the nervous system. Neuromethods.

[CR15] Eng LF, Ghirnikar RS (1994). GFAP and astrogliosis. Brain Pathol.

[CR16] Goujon C, Rivière L, Jarrosson-Wuilleme L, Bernaud J, Rigal D, Darlix JL, Cimarelli A (2007). SIVSM/HIV-2 Vpx proteins promote retroviral escape from a proteasome-dependent restriction pathway present in human dendritic cells. Retrovirology.

[CR17] Grabski E, Waibler Z, Schüle S, Kloke BP, Sender LY, Panitz S, Cichutek K, Schweizer M, Kalinke U (2011). Comparative analysis of transduced primary human dendritic cells generated by the use of three different lentiviral vector systems. Mol Biotechnol.

[CR18] Gundersen HJ, Jensen EB (1987). The efficiency of systematic sampling in stereology and its prediction. J Microsc.

[CR19] Haack K, Cockrell AS, Ma H, Israeli D, Ho SN, McCown TJ, Kafri T (2004). Transactivator and structurally optimized inducible lentiviral vectors. Mol Ther.

[CR20] Harding AJ, Halliday GM, Cullen K (1994). Practical considerations for the use of the optical disector in estimating neuronal number. J Neurosci Meth.

[CR21] Heinzinger NK, Bukrinsky MI, Haggerty SA, Ragland AM, Kewalramani V, Lee MA, Gendelman HE, Ratner L, Stevenson M, Emerman M (1994). The Vpr protein of human immunodeficiency virus type 1 influences nuclear localization of viral nucleic acids in nondividing host cells. P Natl Acad Sci USA.

[CR22] Hocquemiller M, Giersch L, Audrain M, Parker S, Cartier N (2016). Adeno-associated virus-based gene therapy for CNS diseases. Hum Gene Ther.

[CR23] Jakobsson J, Ericson C, Jansson M, Björk E, Lundberg C (2003). Targeted transgene expression in rat brain using lentiviral vectors. J Neurosci Res.

[CR24] James T, Nonnemacher MR, Wigdahl B, Krebs FC (2016). Defining the roles for Vpr in HIV-1-associated neuropathogenesis. J Neurovirol.

[CR25] Johnson WB, Ruppe MD, Rockenstein EM, Price J, Sarthy VP, Verderber LC, Mucke L (1995). Indicator expression directed by regulatory sequences of the glial fibrillary acidic protein (GFAP) gene: In vivo comparison of distinct GFAP-lacZ transgenes. Glia.

[CR26] Kato S, Kobayashi K, Inoue KI, Kuramochi M, Okada T, Yaginuma H, Morimoto K, Shimada T, Takada M, Kobayashi K (2011). A lentiviral strategy for highly efficient retrograde gene transfer by pseudotyping with fusion envelope glycoprotein. Hum Gene Ther.

[CR27] Kloke BP, Schüle S, Mühlebach MD, Wolfrum N, Cichutek K, Schweizer M (2010). Functional HIV-2 and SIVsmmPBj-derived lentiviral vectors generated by a novel polymerase chain reaction-based approach. J Gene Med.

[CR28] Liehl B, Hlavaty J, Moldzio R, Tonar Z, Unger H, Salmons B, Günzburg WH, Renner M (2007). Simian immunodeficiency virus vector pseudotypes differ in transduction efficiency and target cell specificity in brain. Gene Ther.

[CR29] Lundberg C, Björklund T, Carlsson T, Jakobsson J, Hantraye P, Déglon N, Kirik D (2008). Applications of lentiviral vectors for biology and gene therapy of neurological disorders. Curr Gene Ther.

[CR30] Mangeot PE, Duperrier K, Nègre D, Boson B, Rigal D, Cosset FL, Darlix JL (2002). High levels of transduction of human dendritic cells with optimized SIV vectors. Mol Ther.

[CR31] Mashiba M, Collins KL (2012). Molecular mechanisms of HIV immune evasion of the innate immune response in myeloid cells. Viruses.

[CR32] Mitrophanous K, Yoon S, Rohll J, Patil D, Wilkes F, Kim V, Kingsman S, Kingsman A, Mazarakis N (1999). Stable gene transfer to the nervous system using a non-primate lentiviral vector. Gene Ther.

[CR33] Mittermeyer G, Christine CW, Rosenbluth KH, Baker SL, Starr P, Larson P, Kaplan PL, Forsayeth J, Aminoff MJ, Bankiewicz KS (2012). Long-term evaluation of a phase 1 study of AADC gene therapy for Parkinson´s disease. Hum Gene Ther.

[CR34] Moldzio R, Radad K, Duvigneau JC, Kranner B, Krewenka C, Piskernik C, Rausch WD (2006). Glutamate-induced cell death and formation of radicals can be reduced by lisuride in mesencephalic primary cell culture. J Neural Transm.

[CR35] Morris KV, Gilbert J, Wong-Staal F, Gasmi M, Looney DJ (2004). Transduction of cell lines and primary cells by FIV-packaged HIV vectors. Mol Ther.

[CR36] Mühlebach MD, Wolfrum N, Schüle S, Tschulena U, Sanzenbacher R, Flory E, Cichutek K, Schweizer M (2005). Stable transduction of primary human monocytes by simian lentiviral vector PBj. Mol Ther.

[CR37] Naldini L, Blömer U, Gage FH, Trono D, Verma IM (1996). Efficient transfer, integration, and sustained long-term expression of the transgene in adult rat brains injected with a lentiviral vector. P Natl Acad Sci USA.

[CR38] Naldini L, Blömer U, Gallay P, Ory D, Mulligan R, Gage FH, Verma IM, Trono D (1996). In vivo gene delivery and stable transduction of nondividing cells by a lentiviral vector. Science.

[CR39] Palfi S, Gurruchaga JM, Ralph GS, Lepetit H, Lavisse S, Buttery PC, Watts C, Miskin J, Kelleher M, Deeley S, Iwamuro H, Lefaucheur JP, Thiriez C, Fenelon G, Lucas C, Brugières P, Gabriel I, Abhay K, Drouot X, Tani N, Kas A, Ghaleh B, Le Corvoisier P, Dolphin P, Breen DP, Mason S, Guzman NV, Mazarakis ND, Radcliffe PA, Harrop R, Kingsman SM, Rascol O, Naylor S, Barker RA, Hantraye P, Remy P, Cesaro P, Mitrophanous KA (2014). Long-term safety and tolerability of ProSavin, a lentiviral vector-based gene therapy for Parkinson’s disease: a dose escalation, open-label, phase 1/2 trial. The Lancet.

[CR40] Pancio HA, Vander Heyden N, Ratner L (2000). The C-terminal proline-rich tail of human immunodeficiency virus type 2 Vpx is necessary for nuclear localization of the viral preintegration complex in nondividing cells. J Virol.

[CR41] Paxinos G, Franklin KBJ (2003). The mouse brain in stereotactic coordinates: Compact second edition.

[CR42] Piersanti S, Astrologo L, Licursi V, Costa R, Roncaglia E, Gennetier A, Ibanes S, Chillon M, Negri R, Tagliafico E, Kremer EJ, Saggio I (2013). Differentiated neuroprogenitor cells incubated with human or canine adenovirus, or lentiviral vectors have distinct transcriptome profiles. PLoS ONE.

[CR43] Porlan E, Martí-Prado B, Consiglio A, Fariñas I (2016). Stable and efficient genetic modification of cells in the adult mouse V-SVZ for the analysis of neural stem cell autonomous and non-autonomous effects. J Vis Exp.

[CR44] Schoderboeck L, Riad S, Bokor AM, Wicky HE, Strauss M, Bostina M, Oswald MJ, Empson RM, Hughes SM (2015). Chimeric rabies SADB19-VSVg-pseudotyped lentiviral vectors mediate long-range retrograde transduction from the mouse spinal cord. Gene Ther.

[CR45] Schüle S, Kloke BP, Kaiser JK, Heidmeier S, Panitz S, Wolfrum N, Cichutek K, Schweizer M (2009). Restriction of HIV-1 replication in monocytes is abolished by Vpx of SIVsmmPBj. PLoS One.

[CR46] Schulze W, Hayata-Takano A, Kamo T, Nakazawa T, Nagayasu K, Kasai A, Seiriki K, Shintani N, Ago Y, Farfan C, Hashimoto R, Baba A, Hashimoto H (2015). Simultaneous neuron- and astrocyte-specific fluorescent marking. Biochem Bioph Res Co.

[CR47] Singhal PK, Kumar PR, Subba Rao MRK, Kyasani M, Mahalingam S (2006). Simian immunodeficiency virus Vpx is imported into the nucleus via importin alpha-dependent and -independent pathways. J Virol.

[CR48] Sinnayah P, Lindley TE, Staber PD, Cassell MD, Davidson BL, Davisson RL (2002). Selective gene transfer to key cardiovascular regions of the brain: comparison of two viral vector systems. Hypertension.

[CR49] Slomianka L, West MJ (2005). Estimators of the precision of stereological estimates: an example based on the CA1 pyramidal cell layer of rats. Neuroscience.

[CR50] Srivastava S, Swanson SK, Manel N, Florens L, Washburn MP, Skowronski J (2008). Lentiviral Vpx accessory factor targets VprBP/DCAF1 substrate adaptor for cullin 4 E3 ubiquitin ligase to enable macrophage infection. PLoS Pathog.

[CR51] Sterio DC (1984). The unbiased estimation of number and sizes of arbitrary particles using the disector. J Microsc.

[CR52] Stewart HJ, Ralph GC, Fong-Wong L, Strickland I, McClockey L, Barnes L, Blount I, Wells O, Truran CJM, Kingsman AJ, Palfi S, Mitrophanous KA (2016). Optimizing transgene configuration and protein fusions to maximize dopamine production for the gene therapy of Parkinson´s disease. Human. Gene Ther.

[CR53] Stoykova AS, Dabeva MD, Dimova RN, Hadjiolov AA (1985). Ribosome Biogenesis and Nucleolar Ultrastructure in Neuronal and Oligodendroglial Rat Brain Cells. J Neurochem.

[CR54] Trabalza A, Georgiadis C, Eleftheriadou I, Hislop JN, Ellison SM, Karavassilis ME, Mazarakis ND (2013). Venezuelan equine encephalitis virus glycoprotein pseudotyping confers neurotropism to lentiviral vectors. Gene Ther.

[CR55] Vogel R, Amar L, Thi AD, Saillour P, Mallet J (2004). A single lentivirus vector mediates doxycycline-regulated expression of transgenes in the brain. Hum Gene Ther.

[CR56] West MJ (1999). Stereological methods for estimating the total number of neurons and synapses: issues of precision and bias. Trends Neurosci.

[CR57] West MJ, Slomianka L, Gundersen HJ (1991). Unbiased stereological estimation of the total number of neurons in the subdivisions of the rat hippocampus using the optical fractionator. Anat Rec.

[CR58] Wolfrum N, Mühlebach MD, Schüle S, Kaiser JK, Kloke BP, Cichutek K, Schweizer M (2007). Impact of viral accessory proteins of SIVsmmPBj on early steps of infection of quiescent cells. Virology.

[CR59] Wong LF, Goodhead L, Prat C, Mitrophanous KA, Kingsman SM, Mazarakis ND (2006). Lentivirus-mediated gene transfer to the central nervous system: therapeutic and research applications. Hum Gene Ther.

[CR60] Zhang Y, Wang H, Pan H, Bao X, Li M, Jin J, Wu X (2006). Gene delivery into primary cerebral cortical neurons by lentiviral vector. Cell Biol Int.

[CR61] Zufferey R, Donello JE, Trono D, Hope TJ (1999). Woodchuck hepatitis virus posttranscriptional regulatory element enhances expression of transgenes delivered by retroviral vectors. J Virol.

