# Supplementary figures and images for: Tropism, intracerebral distribution, and transduction efficiency of HIV- and SIV-based lentiviral vectors after injection into the mouse brain: a qualitative and quantitative in vivo study
Source: Histochem Cell Biol. 2017 Apr 10;148(3):313–29. doi: 10.1007/s00418-017-1569-1 (PMC5539277; doi:10.1007/s00418-017-1569-1)

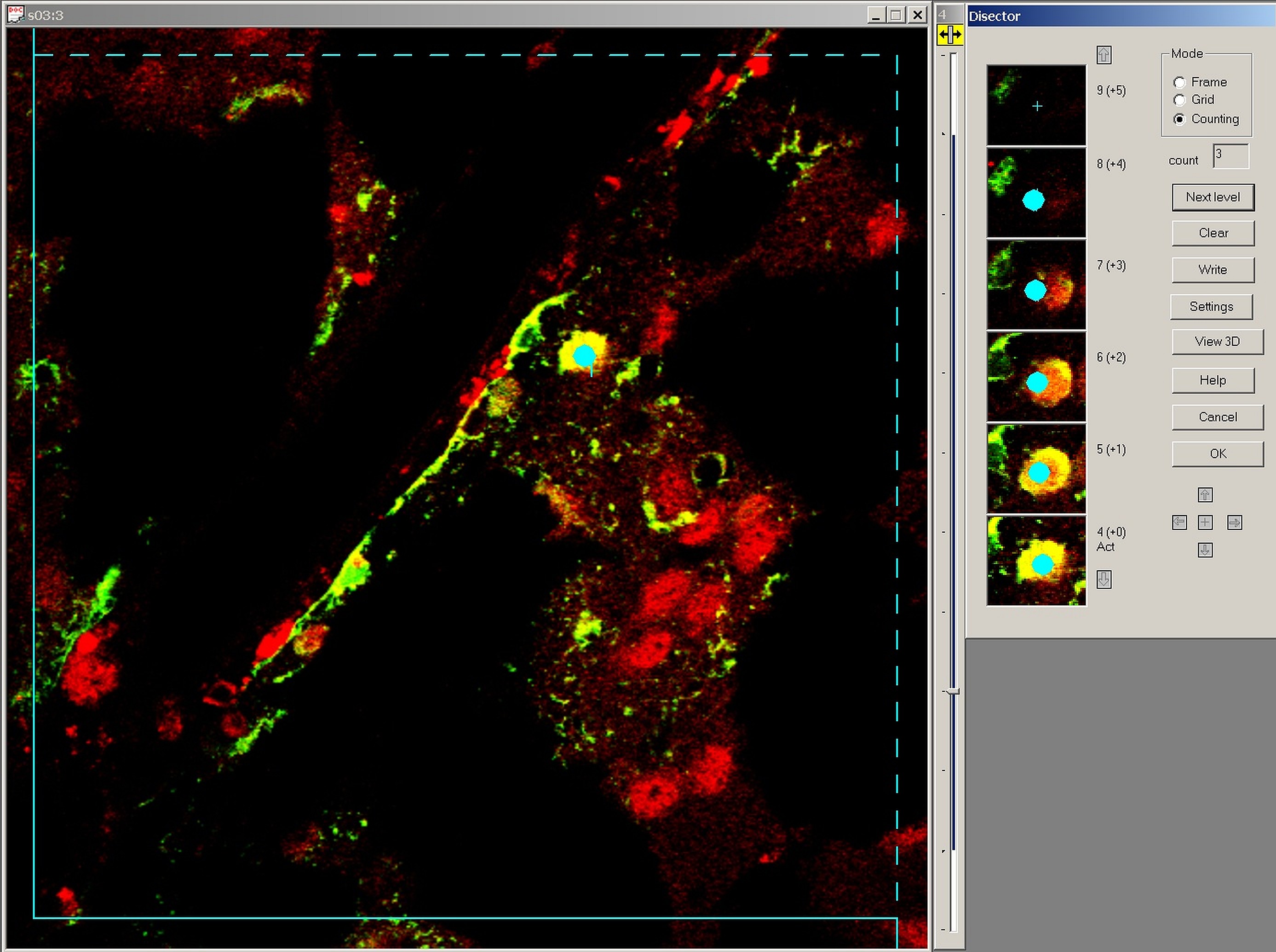

Supplement: Supplementary file 1 — Supplementary material 1 (JPG 572 KB) [file 418_2017_1569_MOESM1_ESM.jpg]

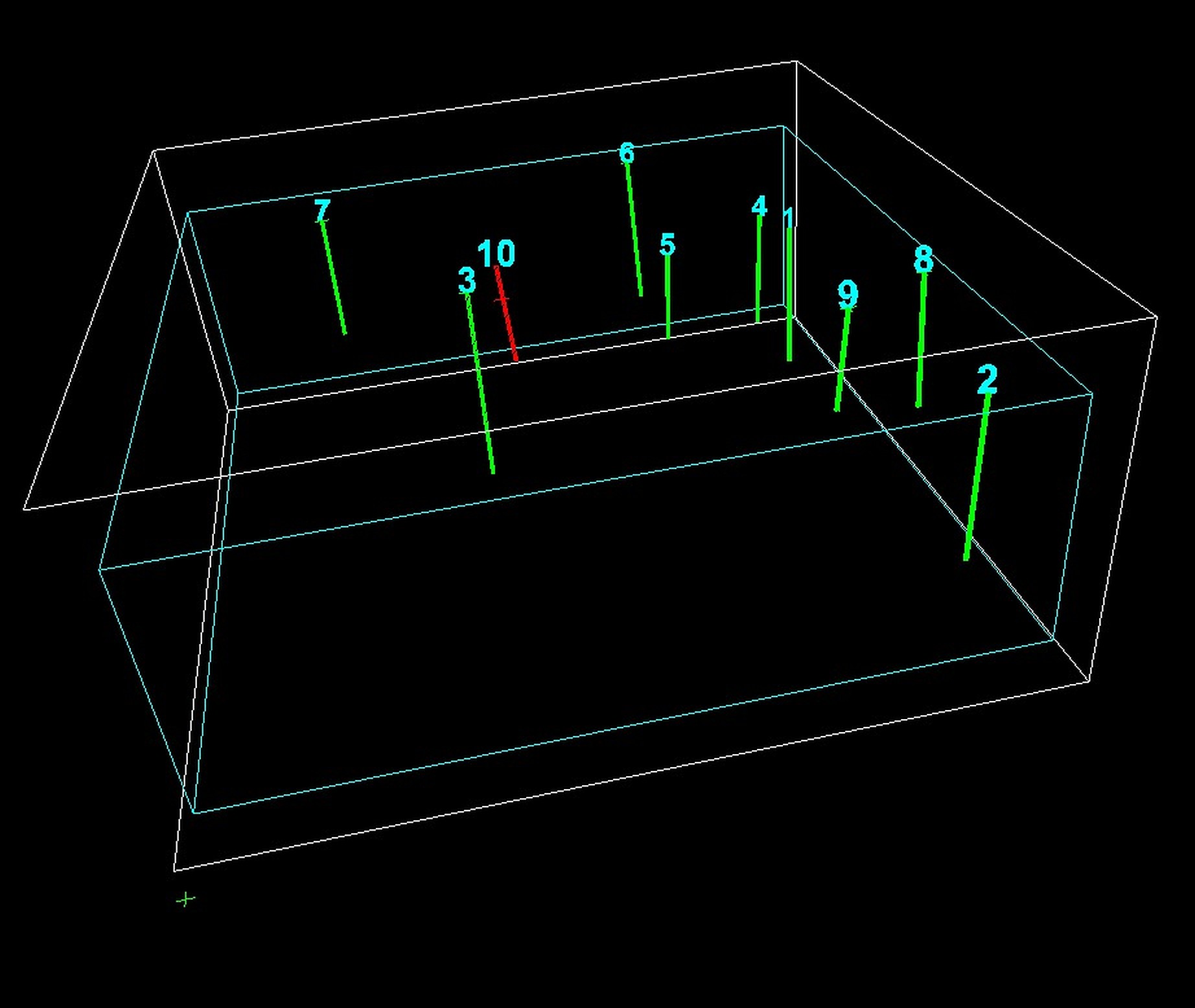

Supplement: Supplementary file 2 — Supplementary material 2 (JPG 187 KB) [file 418_2017_1569_MOESM2_ESM.jpg]

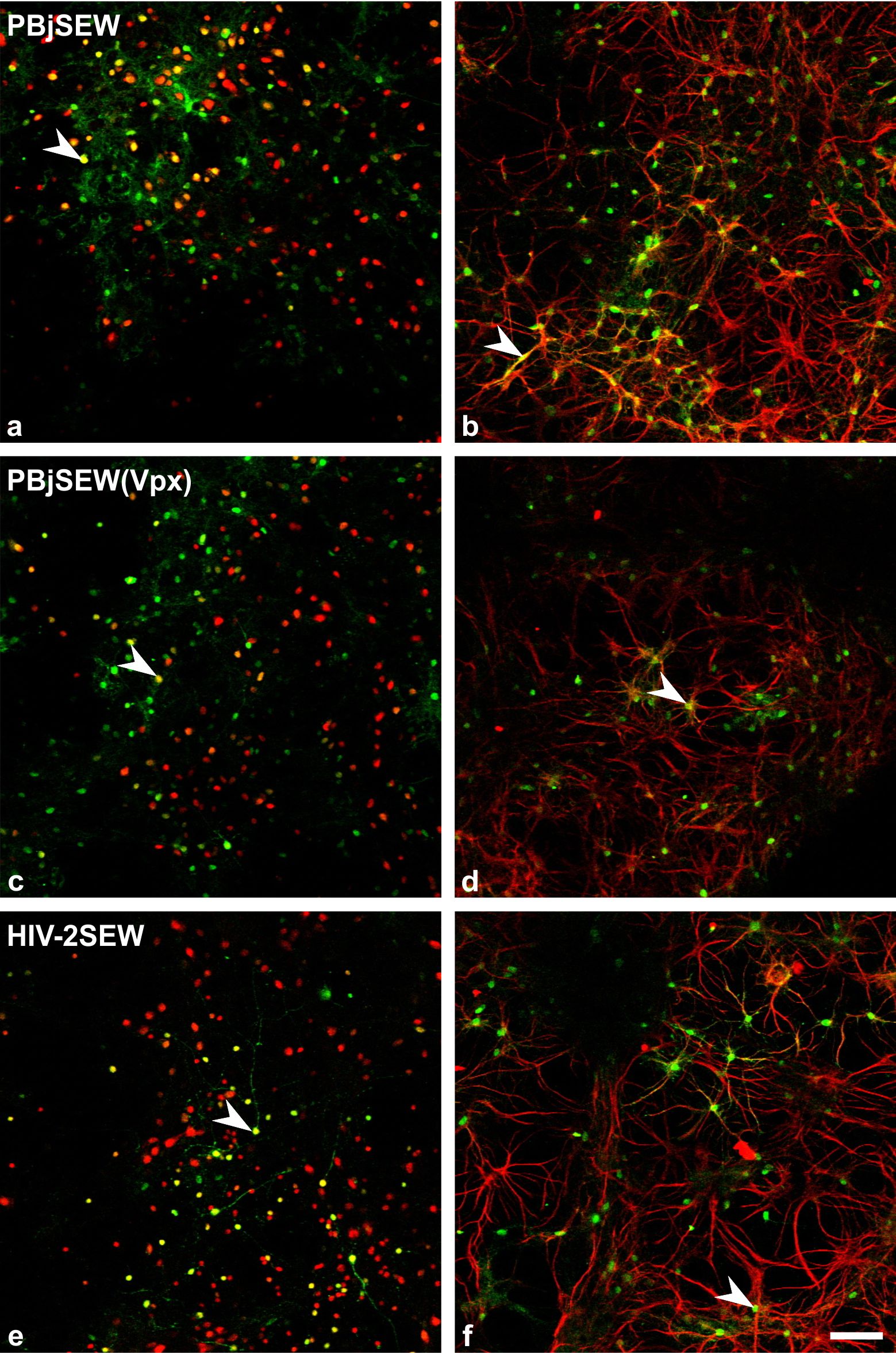

Supplement: Supplementary file 3 — Supplementary material 3 (JPG 520 KB) [file 418_2017_1569_MOESM3_ESM.jpg]

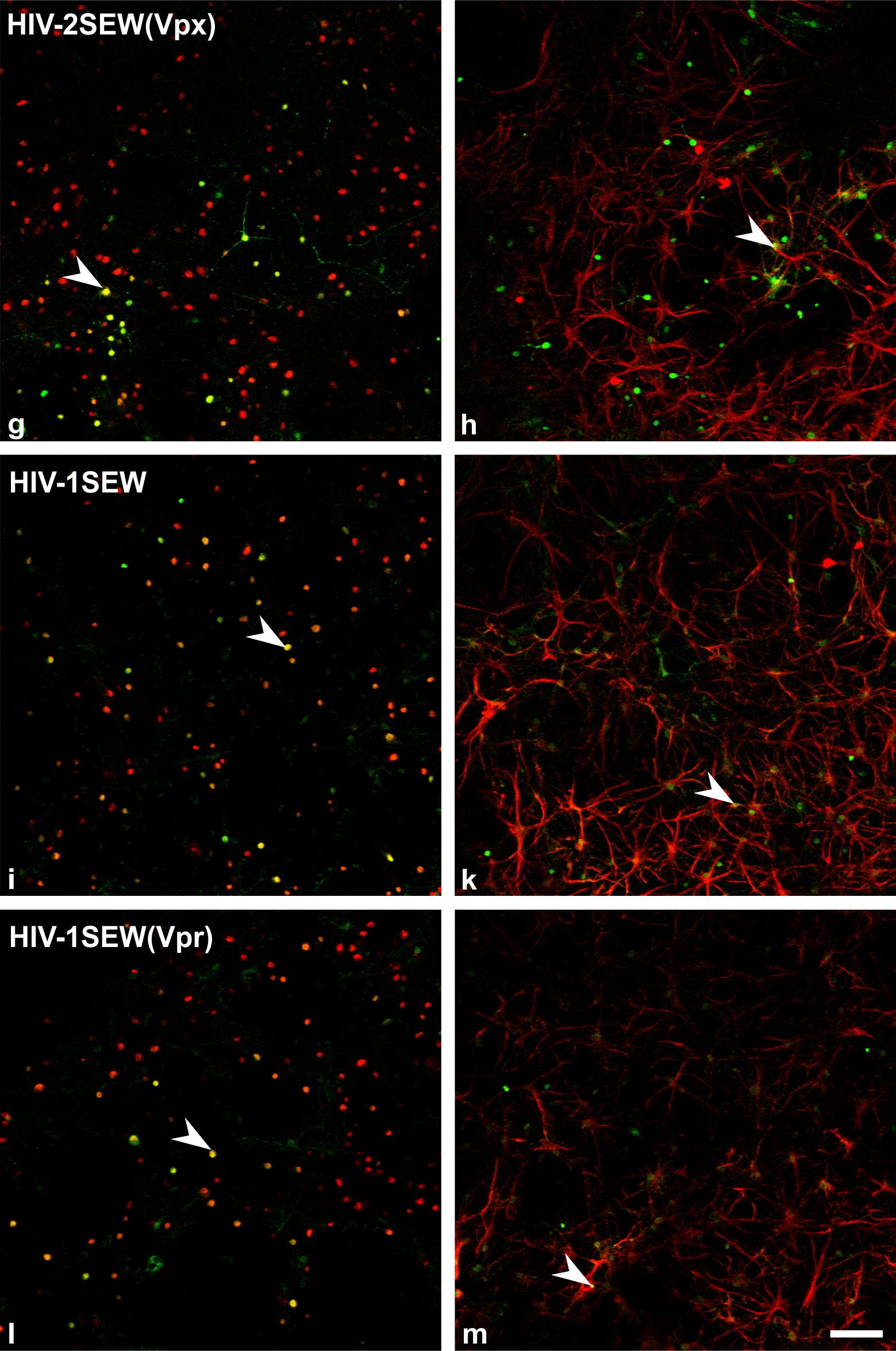

Supplement: Supplementary file 4 — Supplementary material 4 (JPG 394 KB) [file 418_2017_1569_MOESM4_ESM.jpg]

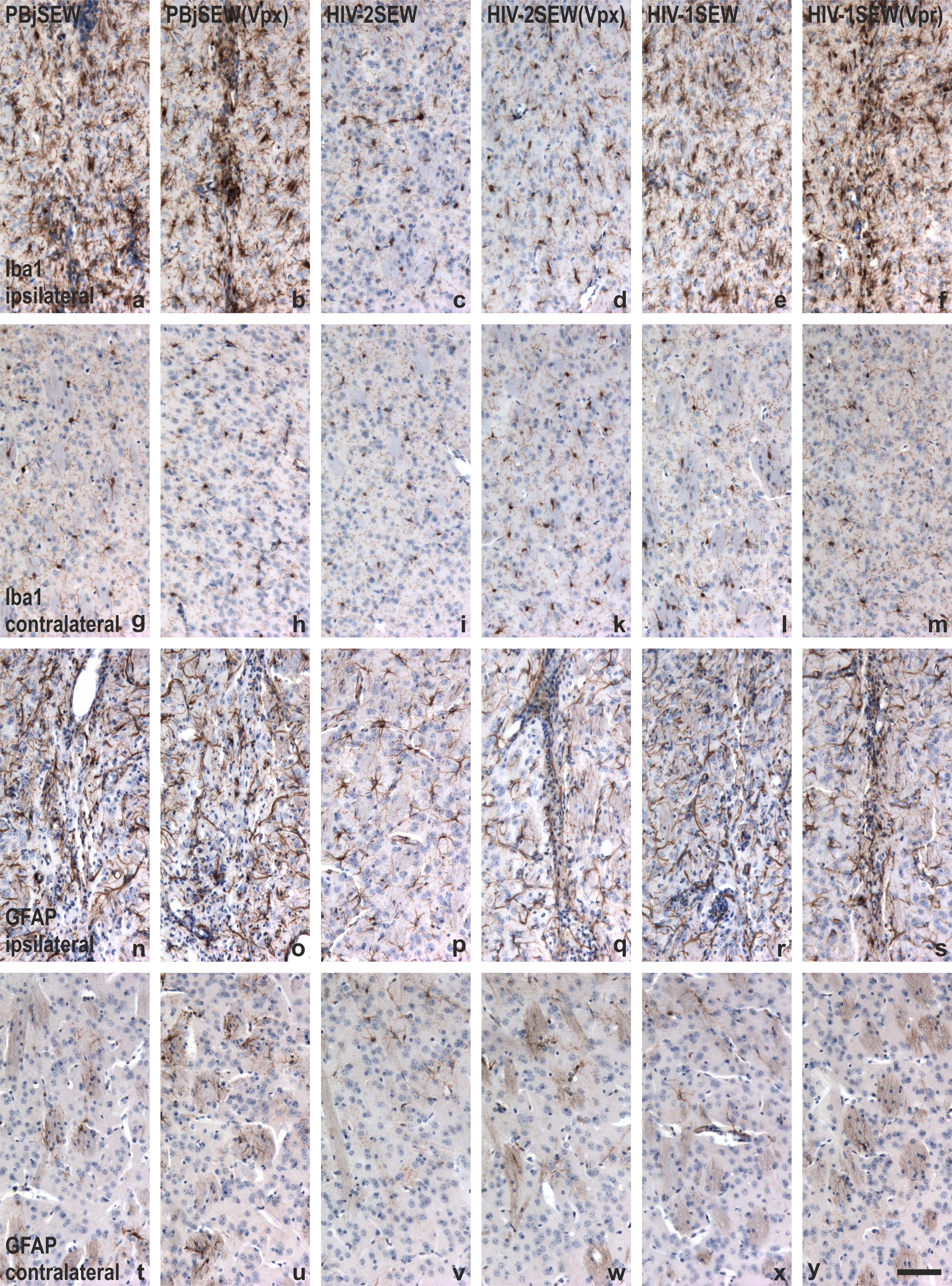

Supplement: Supplementary file 6 — Supplementary material 6 (JPG 5347 KB) [file 418_2017_1569_MOESM6_ESM.jpg]
