# Supplementary material for: Tropism, intracerebral distribution, and transduction efficiency of HIV- and SIV-based lentiviral vectors after injection into the mouse brain: a qualitative and quantitative in vivo study
Source: Histochem Cell Biol. 2017 Apr 10;148(3):313–29. doi: 10.1007/s00418-017-1569-1 (PMC5539277; doi:10.1007/s00418-017-1569-1)
Supplement: Supplementary file 5 — Supplementary material 5 (DOCX 14 KB) [file 418_2017_1569_MOESM5_ESM.docx]

**Online Resource 4** *In-vitro* transduction efficiency for neurons expressed as percentage of EGFP-positive neurons related to total number of neurons as counted in four randomly selected image fields in two infection replicates *per* virus.

| Vector name | % infected  NeuN+ cells | Mean % infected NeuN+ cells |
| --- | --- | --- |
| **PBjSEW** | 40 | **37** |
|  | 34 |  |
| **PBjSEW(Vpx)** | 22 | **20** |
|  | 17 |  |
| **HIV-2SEW** | 39 | **37** |
|  | 34 |  |
| **HIV-2SEW(Vpx)** | 33 | **32** |
|  | 30 |  |
| **HIV-1SEW** | 33 | **31** |
|  | 29 |  |
| **HIV-1SEW(Vpr)** | 20 | **26** |
|  | 31 |  |
